# Innovative treatment for pes cavovarus: a pilot study of 13 children

**DOI:** 10.1080/17453674.2018.1486525

**Published:** 2018-06-18

**Authors:** Ignacio Sanpera Jr, Guillem Frontera-Juan, Julia Sanpera-Iglesias, Laura Corominas-Frances

**Affiliations:** 1 Pediatric Orthopedic Department, Hospital Universitari Son Espases, Palma de Mallorca, Spain;; 2 Research Unit, Hospital Universitari Son Espases, Spain;; 3 Croydon University Hospital, Croydon, UK

## Abstract

Background and purpose — Pes cavovarus (PCV) is a complex deformity, frequently related to neurological conditions and associated with foot pain, callosities, and walking instability. The deformity has the tendency to increase during growth. Orthotic treatment is ineffective and surgery may be troublesome. We present the preliminary results of a new mini-invasive surgical technique for correction of this foot deformity.

Patients and methods — We operated on 13 children (24 feet), age 7–13 years. In 7 children the deformity was neurological in origin. The surgical technique included a dorsal hemiepiphysiodesis of the 1st metatarsal, and a plantar fascia release. The clinical deformity, hindfoot flexibility, and foot callosities, together with a radiological assessment (Meary angle, calcaneal pitch, and talo-calcaneal angle), was done pre- and postoperatively. At final check-up, after a median of 28 months (12–40), the Oxford Ankle Foot Questionnaire for children (OXAFQ-C) was used to assess patient satisfaction. The primary outcome was the hindfoot varus correction.

Results — All the operated feet improved clinically and radiologically. Heel varus improved from a mean 6° preoperatively to 5° valgus postoperatively. In those children where treatment was initiated at a younger age, full correction was achieved. Footwear always improved.

Interpretation — This treatment may offer a less aggressive alternative in the treatment of PCV in young children and may eventually reduce the amount of surgery needed in the future.

Pes cavovarus (PCV) is a complex deformity of the foot produced by the combination of forefoot pronation and inversion of the hindfoot. The etiology of the deformity is reported to be neurological in a majority of cases; Charcot–Marie–Tooth (CMT), a hereditary motor and sensory neuropathy, being the most common cause. This condition is characterized by foot weakness and deformity (Pareyson and Marchesi 2009).

The foot deformity is the result of unbalanced forces applied on a growing foot, leading to a progressive deformity. Although unanimous agreement does not exist, the initial event, at least in Charcot–Marie–Tooth, appears to be denervation of the intrinsic muscles of the foot, resulting in clawing toes and an increase in the height of the foot’s arch, leading to an equinus deformity of the forefoot over the hindfoot. Once the deformity is established it tends to progress. The rate of progression and severity will ultimately depend on the form of Charcot–Marie–Tooth, its causative gene and the type of mutation (Pareyson and Marchesi 2009, Burns et al. 2012). The deformity tends to appear at the beginning of the second decade of life; the heel varus is initially flexible, but in most patients will progress and becomes stiff by the end of the same decade (Aktas and Sussman 2000). The deformity results in foot pain, callosities, ankle instability, and difficulties with footwear (Burns et al. 2009, Wicart 2012, Dreher et al. 2014).

Common treatment for pes cavovarus includes tendon transfers, bone osteotomies located at either the fore- or the hindfoot, and combinations of all of them, determined by the treating surgeon and the severity of the condition.

Hemiepiphysiodesis produces bone reshaping by asymmetrically interfering with bone growth, allowing in this way deformity correction and sparing bone osteotomies. Although the mechanism of application is simple, our understanding of the physiology of this mechanism is limited (Corominas-Frances et al. 2015, Gotliebsen et al. 2016).

The aim of this study was to assess whether the use of dorsal hemiepiphyseal arrest of the 1st metatarsal (1MMT), when combined with a percutaneous fascia plantar release, would stop deterioration or even achieve correction of the hindfoot deformity in the cavovarus foot.

## Patients and methods

During November 2012 to November 2015, 15 children (10 girls) were recruited for a pilot study. This was a proof of concept study to ascertain the effect, safety, and variations that could be expected from the treatment before starting a major trial. In 9 cases the cause of the pes cavovarus was neurological whilst in the remaining 6 children no etiological cause was found. 11 children had bilateral surgery and 4 children were only operated on 1 foot. The reasons behind single foot surgery were: first, asymmetrical involvement (2 feet), and second, previous foot surgery on the contralateral side (2 patients).

The average age for girls at surgery was 10 years (7–13) and for boys 11 years (9–12) for boys.

**Figure 1.**
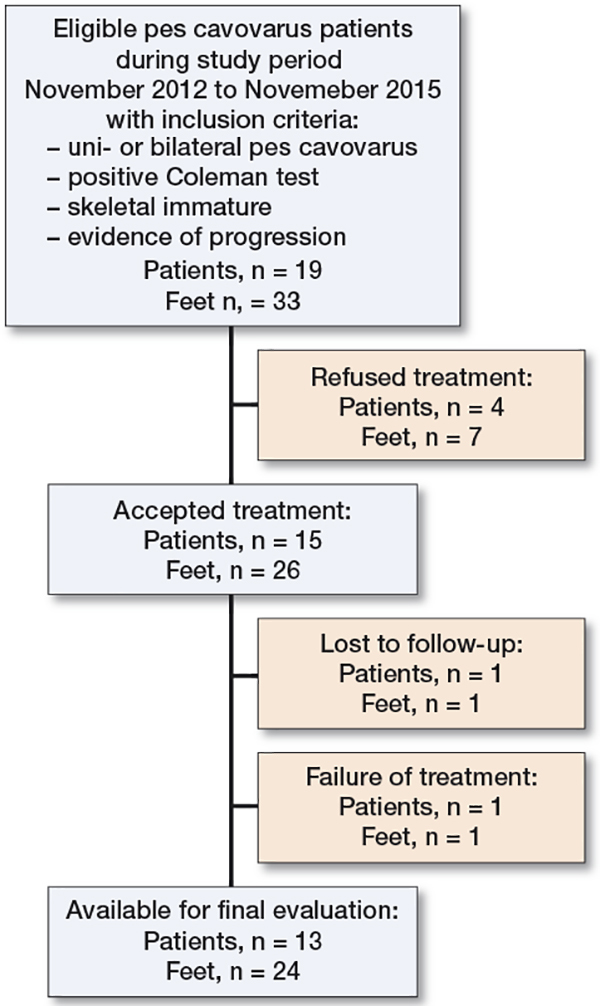
Flowchart of data collection. Inclusion criteria are explained, and number of patients recruited and excluded are detailed.

For the study, we used a number of selection criteria. Patients who fulfilled the criteria were offered the possibility of attending the study and parents signed an informed consent. 4 suitable candidates declined to participate (Figure 1).

**Figure 2.**
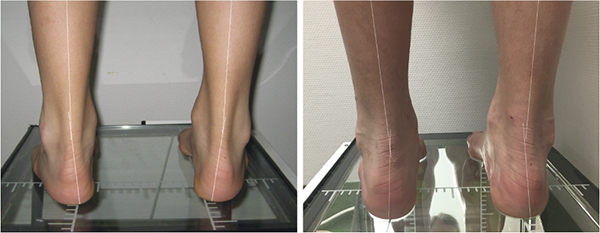
(Left) Preoperative picture of a 9-year-old child standing, seen from the back. The lines drawn show the presence of a heel varus deformity of the hindfoot. (Right) Same child in a similar position, 18 months after surgery. Note the changes in the heel position, now in a valgus position.

**Figure 3.**
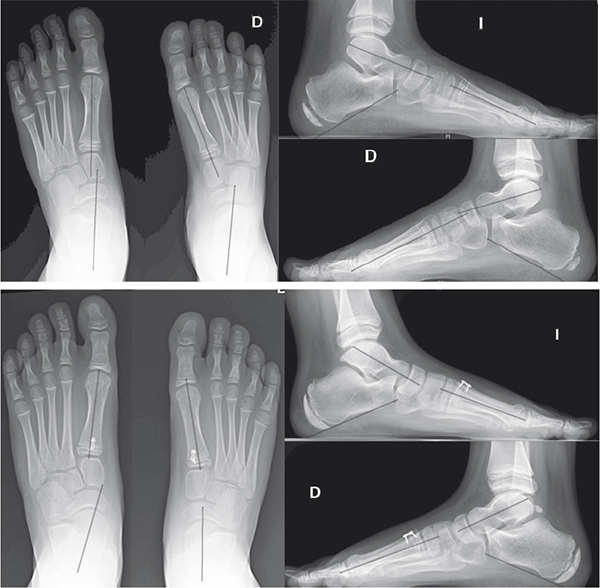
Radiographs of both feet taken preoperatively at 7 years 8 months (top), and postoperatively (bottom, same patient 18 months after surgery), to evaluate correction. On each radiograph the following angles were measured: on the AP standing view, the talus–1st metatarsal angle, and on the lateral standing view Meary’s angle and the calcaneal pitch. Note that the initial involvement was asymmetrical, with the right foot more involved than the left. By 18 months, both feet show signs of hypercorrection.

All patients were assessed both clinically and radiologically. Clinically, the foot deformity was monitored dynamically and statically. Pictures from the back in standing position and during the Coleman block test were taken. The heel angle was measured on the photographs, drawing a line bisecting the heel and a second one bisecting the calf and measuring the angle of confluence (Figure 2). Foot callosities were recorded. Patients and caregivers were questioned about the presence of pain and disabilities in daily life. Preoperative front and lateral standing foot radiographs were obtained in all patients. The following parameters were analyzed: Meary’s angle and calcaneal pitch on the lateral film and 1st metatarsal-talus angle on standing AP view (Figure 3).

The primary outcome was the correction of the heel varus, and the secondary outcome the change in the measured angles and callosities.

At final examination, at a mean of 28 months (12–40) after surgery new clinical photographs were obtained and compared with preoperative images (Figure 2). Radiological values at pre-surgery and at final check-up were also compared (see Figure 3). A foot and ankle questionnaire was handed out to evaluate the extent of disability produced by their foot condition. We used the OXAFQ-C (Morris et al. 2008, 2009), which assesses 3 fields: physical (maximum score 24), school and play (score 16), and emotional (score 16). It also evaluates the limitation that children experience with the use of footwear (maximal score 4). Only 7 patients fulfilled the OXAFQ-C preoperatively, and their responses were compared with the scores achieved at final follow-up.

Finally, patients were specifically questioned about how their footwear usability had changed with surgery, and those who did not complete the OXAFQ-C preoperatively were asked to score, numerically and retrospectively, their footwear difficulty.

Of the 15 patients (26 feet) operated on, 13 patients (24 feet) were available for follow-up (13 of neurological origin and 11 of unknown etiology). 2 patients (2 feet) were lost to follow-up: 1 of them moved away and the second had an unnoticed misplacement of the hemiepiphysiodesis device, resulting in failure.

Additional surgeries included: 2 feet with Jones tendon transfer for symptomatic first toe claw deformity, and 1 foot with a simultaneous tendo Achilles lengthening for a fixed equinus.

As some children did not have same-day surgery on both feet, the follow-up time was referred to feet rather than to patients (in 3 bilateral cases each foot was operated at different stages with an interval range from 3 to 12 months). At the moment of the final check-up 5 patients had already reached skeletal maturity.

## Statistics

Descriptive analysis was performed calculating mean and standard deviation (SD) for continuous variables, with their 95% confidence interval (CI). To measure the effect of hemiepiphysiodesis we calculated the difference between the basal angle and the angle at last follow-up, with their 95% confidence intervals (CI).

As for the foot questionnaire, the issue of unilateral/bilateral involvement was not considered as patients were evaluated as a whole, independent of whether they had single or bilateral foot surgery.

All analyses were performed with IBM SPSS Statistics v.20 (IBM Corp, Armonk, NY, USA).

## Ethics, funding and conflict of interest

Approval from the Local Institution Review Board was obtained, with number CI-185-17 issued on April 1, 2017. The parents of the children signed an informed consent to enter the study. No funding was obtained. There are no conflicts of interest to declare.

## Surgical technique

**Figure 4.**
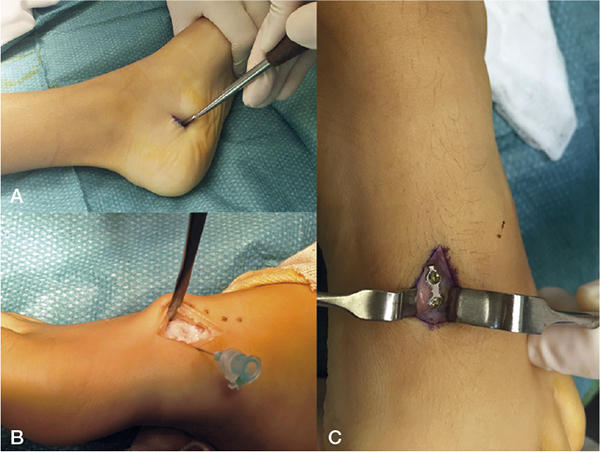
Operative pictures. A: Incision for the percutaneous plantar release. B: A dorsal incision for insertion of the plate is made; the physis is located with the help of a needle and confirmed under an image intensifier. C: detail of the inserted plate.

At the level of the internal tuberosity of the os calcis, on the medial foot border, a 2-centimeter incision was made (Figure 4) in an oblique fashion following the inferior border of the calcaneum. The fibers of the plantar fascia and the foot sole muscles were identified by a blunt instrument and percutaneously sectioned. A periosteal elevator was then introduced through the hole and used to disinsert any possible remaining fibers.

Through a dorsal 1.5 cm incision centered over the proximal physis of the 1st metatarsal, the extensor hallucis longus tendon was identified, and retracted medially. Using a 26-gauge needle the physis was located by punction and its position confirmed with fluoroscopy. Next, a 2-hole plate and screws were inserted bridging the physis, with the proximal screw placed first because is the most technically demanding (1.5 mm diameter) and once the position was ascertained the second screw was place. Finally, the position of the device was confirmed under fluoroscan in both anteroposterior and lateral positions. When in doubt an oblique view was used.

A below-knee plaster was applied with the forefoot in maximal dorsiflexion over the hindfoot and was maintained for a period of 4 weeks. Full weight-bearing was allowed as soon as the patient felt comfortable.

## Results

3 patients had surgical complications. In 2 feet the proximal screw was wrongly placed and needed new surgery to reposition the screw. 1 foot had a rupture of the plate.

**Figure 5.**
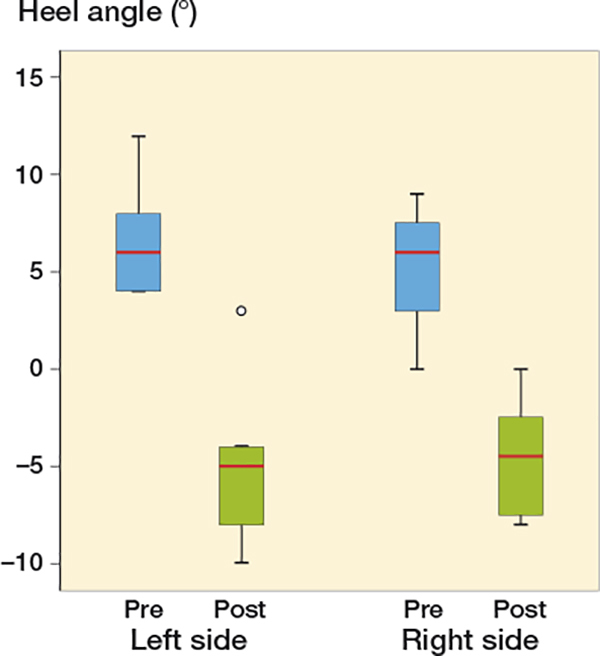
Box plot showing the evolution of the clinical varus deformity after the surgery. The heel angle is reflected at 2 different periods in time, preoperatively (blue) and at final follow-up (green). The results are show by side of foot involvement (please note that positive values in the heel angle line represents varus deformity at the heel, while negative values represent valgus). All the operated feet decreased their varus deformity. The red lines reflect the median heel angle for each group. The bottom of the box represents the 25th percentile and the upper the 75 percentile. The T lines extending from the boxes are the innerfences or whiskers and extend 1.5 the heigth of the box or if no case/row has a value in this range to the minimum and maximum values. The point represents an outlier.

Clinical assessment showed a preoperative mean varus deformity of 6°, which reversed to a mean 5° valgus of the hindfoot. Postoperatively, only one patient had a residual varus, shifting from an initial 12° varus to 3° varus. Altogether the mean correction achieved was 10° (CI 7.6–11) (Figure 5).

At the final follow-up examination, all the callosities had disappeared, except in one patient where its size had considerably shrunk.

All the radiological parameters improved postoperatively (Table 1).

**Table 1.** Changes observed in the different radiologically measured angles preoperatively and at final follow-up

	Mean difference	95% CI
Right foot		
Change in Meary’s angle	–6.8	–9.4 to –4.5
Change in talus–1st metatarsal angle	–8.2	–12.5 to –4.0
Change in calcaneal pitch	–3.6	–5.8 to –1.4
Left foot		
Change in Meary’s angle	–7.1	–10.3 to –4.4
Change in talus–1st metatarsal angle	–9.2	–16.3 to –3.7
Change in calcaneal pitch	–2.2	–4.5 to 0.2

Results are presented according to the side of involvement.

The OXAFQ-C at final follow-up showed an average score of 54 over a maximum of 60. Of the 7 children with available OXAFQ-C preoperatively, all improved (Table 2).

**Table 2.** Results of foot questionnaire: comparison between the different domains of the 7 patients who completed a questionnaire preoperatively and at final follow-up

	Mean score	Mean score	Mean	
Factor	preoperatively	postoperatively	difference	95 % CI
Physical domain	15	21	–5.9	–9.2 to –2.5
School and play	9	13	–3.4	–7.4 to –0.6
Emotional	12	14	–2.4	–5.2 to 0.4

The footwear usability changed from a preoperative average of 1.3 to a postoperative average of 3.5 out of 4 possible (no difficulties).

## Discussion

The cavovarus foot is not strictly a diagnosis, but rather the description of a deformity secondary to different etiologies and with diverse pathomechanics, which makes its treatment complex (Paulos et al. 1980, Mosca 2001, Ward et al. 2008, Wicart 2012). Even in the same individuals, foot involvement can be highly variable and therefore requires an individualized approach for each foot (Burns et al. 2012).

Few reports have dealt with the treatment of PCV at a young age. Due to its natural course, it is at this stage where intervention stands a better chance of being successful.

Most authors agree that non-operative treatment has no role in this condition (Paulos et al. 1980, Burns et al. 2010), other than for symptomatic relief, and surgery is nearly always indicated. However, there are no clear indications for surgery, often leading to unpredictable results.

Pes cavovarus should be considered, at least in the initial stages, as a forefoot problem with adaptive changes of the midfoot and hindfoot (Paulos et al. 1980, Wicart and Seringe 2006, Mubarak and Van Valin 2009). Many authors have used an osteotomy at the base of the 1st metatarsal to achieve correction of the fall of the 1st metatarsal and consequently the hindfoot varus, with good results (Mubarak and Van Valin 2009). However, others, as the apex of the deformity is located at the medial cuneiform (Mosca 2001), prefer the use of an opening plantar wedge osteotomy at the first cuneiform, despite the fact that the effect over the hindfoot varus may be less obvious (Viehweger et al. 2005). 1st metatarsal osteotomies in children are controversial, as the osteotomy has to be located in the diaphysis to avoid physeal damage, and this may lead to a crooked correction far away from the apex of the deformity.

**Figure 6.**
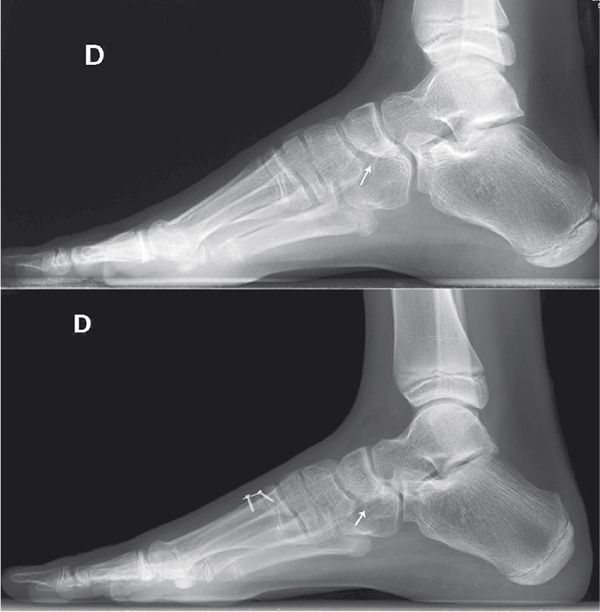
Despite the plate being located at the base of the 1st metatarsal, part of the correction occurs on the surrounding bones. The arrow points at the changes seen at the navicular bone in a patient 2 years after surgery. Note the enlargement of the navicular on its plantar aspect.

Addressing the forefoot deformity at the physis of the 1st metatarsal allows for a progressive correction of the deformity (with the subsequent adaptive changes on the surrounding tissues) and, on the other hand, the correction point is located at close proximity to the apex of the deformity. Moreover, our radiological study suggests that part of the deformity correction happens in the adjacent tarsal bones, which is likely due to the foot pressure changes (Figure 6).

The use of hemiepiphysiodesis of the proximal physis of the 1st metatarsal has previously been successfully used for treatment of hallux valgus. According to the literature, the proximal 1st metatarsal physis closes at around the age of 14.2 years in girls and 15.6 years in boys (Greene et al. 2017). There are 2 reasons for using a temporary rather than a definitive hemiepiphysiodesis. First to be able to reverse the effect in case of overcorrection (Figure 3), and second to have an immediate effect, as opposed to the ablation hemiepiphysiodesis (Ross and Zionts 1997, Stevens 2007).

Muscle balancing is certainly the most difficult part of the treatment of PCV. There is no consistent pattern of muscle imbalance (Mubarak and Van Valin 2009) and evaluation in a young child may be extremely difficult. There is general agreement that muscle imbalance, at least in CMT disease, starts with progressive weakness of the intrinsic foot muscles, resulting in hyperextension of the 1st metatarsophalangeal joint and, consequently, through a windlass effect in a height increase of the longitudinal arch of the foot. Percutaneous plantar release at this initial stage would not only lengthen the short intrinsic muscle, but would also facilitate the hemiepiphysiodesis effect by decreasing the plantar pressures.

Another theoretical advantage of early PCV correction is to avoid the abnormal muscle growth associated with a deformed position. In order for the muscle to grow normally, it requires overstretching of its fibers (Gough and Shortland 2012). If the foot remains in a constant varus position, the tibial muscles will never reach full length, increasing the varus deforming forces at the hindfoot. The opposite would apply to the over-lengthened foot evertors. This might explain why in our cases we have not required further tendon transfers. However, as the condition in most PCVs is progressive, it is likely that further muscle rebalancing will be needed.

Whether the plantar fascial release is responsible on its own for the correction achieved may be discussed. However, multiple reports have shown that although radical plantar release may correct cavus, it is unable to reverse hindfoot varus (Paulos et al. 1980, Sherman and Westin 1981). Furthermore, the role of the hemiepiphysiodesis in valgus correction was clearly proved by our case with a foot deformity relapse after plate breakage.

2 patients underwent 3 additional surgeries. As none of these procedures has proven to correct the hindfoot varus deformity, we did not exclude these patients from the final analysis.

The most frequent complication was proximal screw misplacement. Many reasons could explain this difficulty: the small size of the proximal epiphysis, the oblique position of the physis to the ground, and, last but not least, the superposition of multiple anatomical structures with the proximal physis of the 1st metatarsal on the lateral view radiographs. In 3 feet the screw was incorrectly positioned. In 2 instances the misplacement was detected immediately postoperatively and corrected, but in the remainder it went undetected and determined the failure of the procedure.

1 patient who was operated bilaterally had a relapse in 1 of the feet. Radiographs revealed breakage of the plate. Another possible adverse event is overcorrection of the deformity, which can be observed in younger patients and is a good reason to consider the use of a temporary arrest rather than a definitive one (Figure 3).

The limitations of our study are the number of patients enrolled and the short follow-up. Nevertheless, it is long enough to demonstrate that, with minimal surgery, a normal or near-normal shaped foot can be reached at skeletal maturity. This procedure is not intended to be the definitive treatment for these children as the condition is progressive, and there is actually no effective treatment for most of the causes. Nevertheless, this procedure allows for control of the deformity, and makes the foot less symptomatic, as reflected by the improvement in footwear and the OXAFQ-C.

The use of a material not specifically designed for this type of surgery is another limitation and may explain some of the complications encountered such as plate breakage (due to inadequate trimming of the plate).

In summary, we believe that this technique may be useful for the treatment of pes cavovarus during childhood, although its use should be limited to cavovarus associated with weakness or the idiopathic form. The technique follows the principles laid down by Mosca (2001) for the treatment of pes cavus. First, the segmental deformities should be corrected while preserving joint motion (1st metatarsal hemiepiphysiodesis corrects the fall of the metatarsal). Next, the remaining muscle forces should be balanced out (achieved by plantar fascia release). Third, this leaves open the possibility of reasonable treatment options because the foot remains almost untouched.

Design of the study: IS, GFJ. Data collection: IS, LCF. Data analysis: IS, GFJ, JSI, LCF. Writing the paper: IS, GFJ, JSI

*Acta* thanks Hanne Hedin and Line Kjeldgaard Pedersen for help with peer review of this study.
